# Fitness of Jiaozi starter for steamed bread production using a two‐stage procedure

**DOI:** 10.1002/fsn3.693

**Published:** 2018-06-04

**Authors:** Shiwei Wang, Haifeng Li, Zhijian Li, Yanmei Sun, Jinshui Wang, Meng Li

**Affiliations:** ^1^ Beijing Advanced Innovation Center for Food Nutrition and Human Health Beijing Technology and Business University (BTBU) Beijing China; ^2^ College of Bioengineering Henan University of Technology Zhengzhou China; ^3^ College of Food Science and Technology Henan University of Technology Zhengzhou China; ^4^ School of Chemical Engineering and Energy Technology Dongguan University of Technology Dongguan Shi Guangdong Sheng China; ^5^ Beijing Key Laboratory of Plant Resources Research and Development Beijing Technology and Business University (BTBU) Beijing China

**Keywords:** CO_2_ production and retention, diversity of bacteria and fungi, dough fermentation, Jiaozi starter, reducing sugar utilization, steamed bread

## Abstract

Steamed bread is a popular staple food in China. Jiaozi shows many advantages as a starter for dough fermentation and is frequently used for steamed bread production. The knowledge about the dough fermentation process using Jiaozi is helpful for production management and quality improvement of the final product. In this study, the applicability of Jiaozi for steamed bread production was investigated. Some important factors involved in dough fermentation were carefully examined and analyzed, including the evolution and diversity of major bacteria and fungi, acidity change, reducing sugar utilization, CO_2_ production and retention, and different full dough fermentation periods. Combined with the quality evaluation of the final product, the results displayed that traditional Jiaozi was suitable as starter for steamed bread production using a two‐stage procedure with a wide range of full fermentation time and also provided more insights into steamed bread production by Jiaozi.

## 1 INTRODUCTION

Chinese steamed bread is an important traditional staple food, which is formulated with wheat flour, water, and starter (Zhu, 2014). In recent years, baker’s yeast is used as a starter in industrially making Chinese steamed bread (Yeh, Wu, Charles, & Huang, 2009). However, steamed bread prepared by yeast lacks the flavor and taste produced by traditional starters, such as Jiaozi and sourdough (Corsetti et al., 1998; Denkova et al., 2014; Li, Li, & Bian, 2016; Torrieri, Pepe, Ventorino, Masi, & Cavella, 2014; Wu et al., 2012). When traditional starter is used for steamed bread production, a two‐stage fermentation procedure was usually adopted. Dough firstly finishes full fermentation, and then, more flour is mixed in with a ratio of about 40% by weight. Thereafter, the remixed dough was sheeted, split into small portions, molded, proofed, and steamed (Li, Deng, Li, Liu, & Bian, 2015).

Full dough fermentation is the critical stage for making steamed bread, and the unique microbial community in traditional starters could contribute to the quality of the full fermentation dough. Jiaozi, generally prepared by maize and rice flour, is quite different from sourdough (Li, Li, Qu, & Wang, 2017). Especially, soda addition is dispensable when Jiaozi is used as the dough starter for steamed bread production because sour quality of steamed bread needs to be avoided (Li et al., 2017). This characteristic was speculated to related to the different microbial community structure and abundance between Jiaozi and sourdough (Li et al., 2016; Luangsakul, Keeratipibul, Jindamorakot, & Tanasupawat, 2009; Zhang et al., 2011). *Pediococcus pentosaceus*,* Lactobacillus plantarum*,* Acetobacter tropicalis,* and *Enterococcus durans* have been found to be the dominant bacteria, and *Saccharomyces cerevisiae*,* Wickerhamomyces anomalus*,* Torulaspora delbrueckii,* and *Saccharomycopsis fibuligera* were the main yeast species in Jiaozi (Li et al., 2016). In sourdough, *L*. *plantarum*,* Leuconostoc citreum*,* Weissella cibaria*,* L. casei*,* S*.* cerevisiae, Candida humilis*,* C. tropicalis*, and *Pichia stipitis* were the predominant microbes (Luangsakul et al., 2009; Zhang et al., 2011). In addition, the ratio of yeast to lactic acid bacteria (LAB) in Jiaozi is ~ 1:1, which is higher than in sourdough with a ratio from about 1:1000 to 1:100 (Li et al., 2016; Zhang et al., 2011).

Recently, the bacterial diversity of Jiaozi and dough during fermentation started by Jiaozi was evaluated by high‐throughput sequencing method. The results revealed that the profiles of microorganisms of Jiaozi and dough were similar when the dough was fermented for 8 hr and 24 hr using Jiaozi starter. The predominant bacteria were some species from *Lactobacillus*,* Weissella*, and *Leuconostoc* in Jiaozi and dough*,* and the species from *Lactobacillus* were dominated in the fermented dough (Li et al., 2017). The similar pattern of bacterial community indicated a better adaptation of microbes to the dough conditions. However, little work has been conducted to investigate the influence of the full dough fermentation on the quality of steamed bread. When a starter is selected to use for dough fermentation in industry, its stability is as important as functional features (Minervini et al., 2010). Understanding the stability of Jiaozi for dough fermentation will be of great help for the production management and improvement of the quality and stability of the final product. In addition, the yeast communities responsible for carbon dioxide (CO_2_) production and flavor formation have not been documented.

In this study, the possible factors involved in steamed bead production by Jiaozi starter during wheat dough fermentation were carefully examined, including the evolution and diversity of bacteria and fungi, acidity change, reducing sugar utilization and CO_2_ production. In addition, the quality of steamed bread prepared by Jiaozi with different full dough fermentation times was also evaluated.

## 2 MATERIALS AND METHODS

### 2.1 Materials

Traditional Jiaozi starter was made from maize flour, muskmelon and Chinese Daqu, and dry powder of Jiaozi was obtained from Shangqiu, Henan, China (Li et al., 2017). White wheat flour with 10.72% protein, 0.36% ash, and 13.89% moisture was supplied by Jinyuan Flour Co., Ltd. (Zhengzhou, China). Other chemical reagents were of analytical grade.

### 2.2 Dough fermentation

Fifty grams of Jiaozi starter was ground into powder and mixed with 500 g of wheat flour and 225 ml of water. After stirring for 13 min in SZM5 mixing machine (Xunzhong Co. Ltd., Guangzhou), the dough was fermented at 35°C and 85% relative humidity for 24 hr in a controlled fermentation cabinet (HWS180; Bilon Instrument Co. Ltd., Zhengzhou, China).

### 2.3 Reducing sugar utilization and acidification

Ten grams of dough samples was collected from the fermented dough and homogenized with 90 ml of distilled water on ice for 15 min. Then, the samples were centrifuged with 5000 r/min at 4°C for 10 min. The supernatant fraction was used for reducing sugar analysis by the 3,5‐dinitrosalicylic acid method, and the standard curve was plotted using various concentrations of maltose (Miller, 1959). Before sugar analysis, a Carrez precipitation was performed to eliminate proteins from the dough samples (Sterr, Weiss, & Schmidt, 2009). In brief, 0.5 ml of Carrez I solution [3.6% (wt/vol) K_4_Fe(CN)_6_∙3H_2_O] was added into 8 ml of samples and incubated at room temperature for 1 min, followed by addition of 0.5 ml Carrez II solution [7.2% (wt/vol) ZnSO_4_∙7H_2_O] and 1 ml of 100 mM NaOH and incubation at room temperature for another 5 min. Then, the samples were centrifuged at 8000 r/min at 4°C for 30 min and the supernatant fractions were used for sugar analysis.

### 2.4 pH and the titratable acid assay (TTA)

The pH and total TTA values of the dough samples were determined as previously described (Edema & Sanni, 2008). Briefly, 10 g of the sample was homogenized with 90 ml of sterile distilled water. The values of pH were recorded and the acidity was titrated with 0.1 N NaOH to a final pH 8.5. The TTA was expressed in milliliter of 0.1 N NaOH.

### 2.5 Microbial enumeration

Numbers of colony‐forming units (CFUs) were determined by classical spread‐plating method on YPD agar plates supplemented with 0.1 g/L chloramphenicol for yeasts and on MRS agar plates containing 0.1 g/L of cycloheximide for LAB, respectively (Li et al., 2016).

### 2.6 CO_2_ production and retention

The Rheofermentometer F3 (Chopin, Villeneuve‐La‐Garenne Cedex, France) was used to examine the total CO_2_ release and retention during fermentation process. Immediately after mixing, 315 g of the dough samples prepared according to Section 2.3 was placed in the fermentation cabinet with an additional 2.0 kg of disk on the top of the dough samples. The proofing cabinet was closed tightly, and dough fermentation was performed at 35°C for 6 hr. The changes in the fermenting dough were measured in terms of CO_2_ production and retention from the dough.

### 2.7 Polymerase chain reaction—denaturing gradient gel electrophoresis (PCR‐DGGE) analysis

PCR‐DGGE analysis was conducted as previously reported (Li et al., 2016). Briefly, after DNA extraction from Jiaozi and dough samples, the V3 region of the 16S rRNA gene of bacteria was amplified using the universal primers (GC‐338F and 518R) (Nakatsu, Torsvik, & Øvreås, 2000). The specific 18S rRNA gene of fungi was amplified by the universal primers (NS1 and GC‐fung) (Hoshino & Morimoto, 2008). The PCR products were separated in an 8% polyacrylamide gel with a 30%–55% urea‐formamide linear denaturing gradient for DGGE analysis of bacteria and 20%–45% denaturing gradient for specific DGGE analysis of fungi. Electrophoresis was conducted in 1× TAE buffer at a constant voltage of 150 V at 60°C for 4 hr for the V3 region of the 16S rRNA gene of bacteria and at a constant voltage of 50 V at 60°C for 20 hr for the 18S rRNA gene of fungi. Following silver staining, the bands were excised, recycled using Poly‐Gel DNA Extraction Kit of OMEGA, and re‐amplified. The identity of the microorganisms was revealed by sequencing selected bands from the profiles of DGGE. The homology comparison was performed using BLAST via the National Center for Biotechnology Information (NCBI) (http://www.ncbi.nlm.nih.gov/BLAST).

### 2.8 Steamed bread production

Two‐stage fermentation method was used for steamed bread production according to the previously described with small modification (Li et al., 2015). Firstly, the dough was prepared according to Section 2.3 and fermented at 35°C and 85% relative humidity for 6 hr–8 hr in a cabinet (HWS‐180, BiLon Instrument Co. Ltd.). Secondly, 200 g of wheat flour and 70 ml of water was added into the dough prepared in the first‐stage and mixed for 12 min. Then, the dough was sheeted 10 times on the surface pressure machine (YT‐350; Yinying Instrument Technology Co. Ltd., Shandong, China) and split into 100 g portions. The chunks were formed into round shape by hand and fermented at 35°C and 85% relative humidity for 50 min. Then, the proofed dough was steamed for 30 min in a pot using a steam tray and boiling water.

The steamed bread was also made by one‐stage fermentation method (a straight dough process) (Yeh et al., 2009). The dough after mixing according to Section 2.3 was sheeted 10 times on the surface pressure machine (YT‐350, Yinying Instrument Technology Co. Ltd.) and split into 100 g portions. The chunks were formed into round shape by hand and fermented at 35°C and 85% relative humidity for 1 hr in a cabinet (HWS‐180, Bilon Instrument Co. Ltd.) and then steamed for 30 min.

### 2.9 Quality evaluation of steamed bread

After cooling at room temperature for 1 hr, the quality of steamed bread was evaluated. Hardness of steamed bread was determined as previously reported using a Texture Analyzer (TA.XT2i; Stable Micro Systems, Ltd., Godalming, UK) (Li et al., 2015). Specific volume of steamed bread was measured using the rape seed displacement method, and the whiteness was determined by whiteness meter (WGB‐IV, Tasan Co. Ltd., Hangzhou, China) (Li et al., 2015; Sim, Noor Aziah, & Cheng, 2011). Sensory evaluation of steamed breads was performed by eight trained panelists. The scores were assigned as follows: surface whiteness, 10; smoothness, 10; crumb whiteness, 5; structure, 10; elasticity, 10; stickiness, 10; softness and cohesiveness, 10; flavor, 25 and sour, 10.

### 2.10 Statistical analysis

The data reported in this article were subjected to analysis of variance (ANOVA) by Duncan’s multiple‐range test (*p < *0.05) using SPSS software (SPSS 19.0, SPSS Inc., Chicago, IL, USA) wherever applicable.

## 3 RESULTS AND DISCUSSION

### 3.1 DGGE profiles of bacteria and fungi

**Figure 1:**
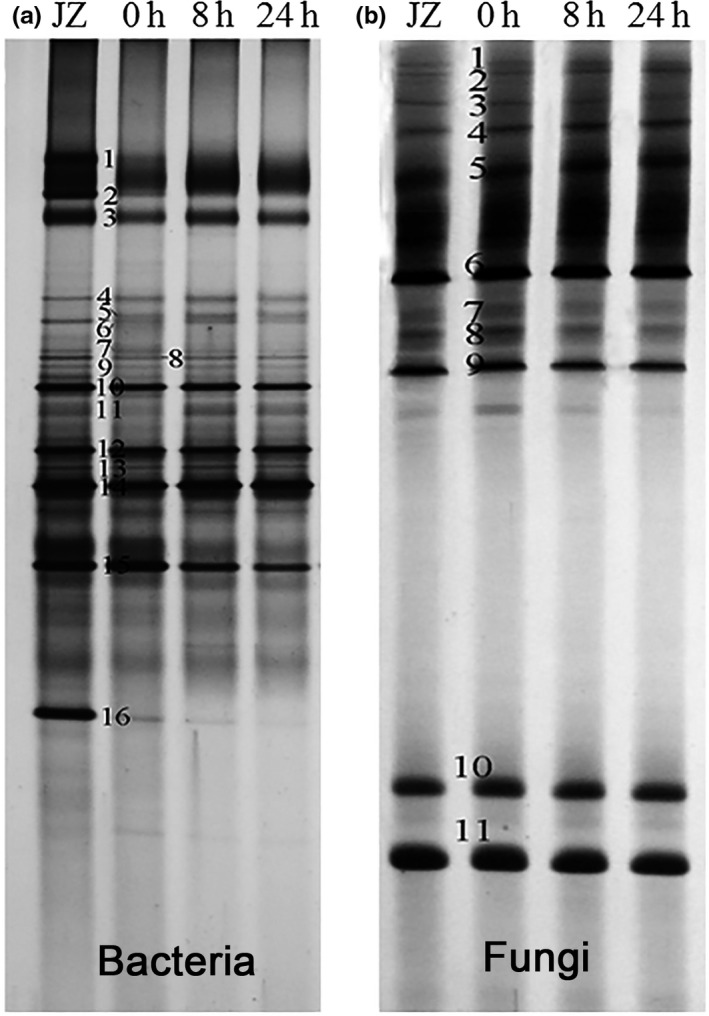
Bacterial (a) and fungal (b) PCR‐DGGE profiles of Jiaozi and fermented dough

The diversity of the major bacteria and fungi associated with dough fermentation using Jiaozi as the starter was analyzed using PCR‐DGGE. The results displayed the PCR‐DGGE profiles of the 16S rRNA and 18S rRNA genes from the microbial community in the starter and fermented dough, and a similar microbial profile was observed for Jiaozi and the fermented dough (Figure 1), indicating that Jiaozi was the main source of the bacteria and fungi in the dough and the dominant microbial populations remained during the fermentation process. A stable bacterial community during dough fermentation started by Jiaozi was also revealed by high‐throughput sequencing method (Li et al., 2017).

Sequencing and identification of the dominant bands displayed the presence of *L*.* plantarum*,* Weissella paramesenteroides*,* Lactobacillus brevis*,* P*.* pentosaceus,* and *Lactobacillus alimentarius* in the Jiaozi starter and fermented dough (Figure 1a and Table 1). The result was consistent with the analysis of high‐throughput sequencing (Li et al., 2017). Some microbes, such as the species belonged to *Lactobacillus*,* Weissella,* and *Pediococcus*, were identified by both methods. However, some discrepancies were also observed. Using the high‐throughput sequencing method, genus *Acetobacter* was found to be predominant in the Jiaozi starter, but it was not detected using the PCR‐DGGE method. This discordance might be because the two analyses based on PCR arise different biases due to PCR amplification selectivity of different primers, and the hypothesis was in accordance with the previous results (Li et al., 2016; Madoroba et al., 2011; Mukisa et al., 2012). The results highlighted the necessity of a combined usage of varied approaches for detection of microbial communities within complex matrices.

**Table 1:** Identities of bands obtained from bacterial and fungal community of Jiaozi and fermented dough

Microbial groups	Band Number	Similar strain	Accession number	Similarity (%)
Bacteria	1	*Triticum turgidum* subsp*. durum* cultivar Langdon chloroplast	KM352501.1	100
	2, 3, 4, 6, 7, 8	*Lactobacillus plantarum*	KR816164.1	100
	5	*Weissella paramesenteroides*	KM392070	100
	9, 15	*Lactobacillus brevis*	KC753454	100
	10, 11, 12, 14	*Pediococcus pentosaceus*	KC753459	99
	13	*Lactobacillus alimentarius*	NR044701	99
	15	*L. brevis*	KC753454	100
	16	*Vigna radiata* var. *sublobata* chloroplast	AP014692.1	100
Fungi	1, 2, 3, 4, 5, 6	*Saccharomyces cerevisiae*	NR132222	99
	7, 8, 9, 11	*Wickerhamomyces anomalus*	KJ659884	99
	10	*Mucor indicus* SDM‐13	AY054699y	100

In addition, band 5 in Figure 1a identified as *W*.* paramesenteroides* in the dough was not detected in the starter (Figure 1a and Table 1), indicating *W*.* paramesenteroides* detected at the dough fermentation stage could originate from the wheat flour. Thus, the initial wheat flour powder could be the potential sources of bacteria for dough fermentation. The hypothesis was consistent with the previous result, which has found wheat flour was an important inoculum for some LAB strains (Alfonzo et al., 2013). PCR‐DGGE analysis showed two common bands in the starter identified as chloroplast DNA from *Triticum turgidum* and *Vigna radiata* var. *Sublobata*, respectively, which might be from the raw materials.

Band identification showed the dominant yeast species to be *S*. *cerevisiae* and *W*. *anomalus,* and they remained stable during dough fermentation (Figure 1b and Table 1). *Saccharomyces cerevisiae* is the most effective CO_2_ and ethanol producer in the fermentation of wheat and maize dough and was frequently reported as being predominant in a variety of foods (De Vuyst, Harth, Van Kerrebroeck, & Leroy, 2016; Greppi et al., 2013). In traditional sourdough fermentation, *W. anomalus* is the second most isolated yeast in sourdough fermentations, and in combination with LAB species, they have been reported to be associated with the formation of flavor and ethanol (Daniel, Moons, Huret, Vrancken, & De Vuyst, 2011; Zheng et al., 2012). The presence of *Mucor indicus* was probably from the raw material used for Jiaozi preparation. The problematic multiple banding pattern that some bacteria and yeasts species were observed could be attributed to the sequence heterogeneities between multiple copies of the 16S rRNA or 18S rRNA gene in any given strain. The observation was in accordance with the previous results (Chao, Huang, Kang, Watanabe, & Tsai, 2013; Madoroba et al., 2011).

### 3.2 Reducing sugar utilization during fermentation

**Figure 2:**
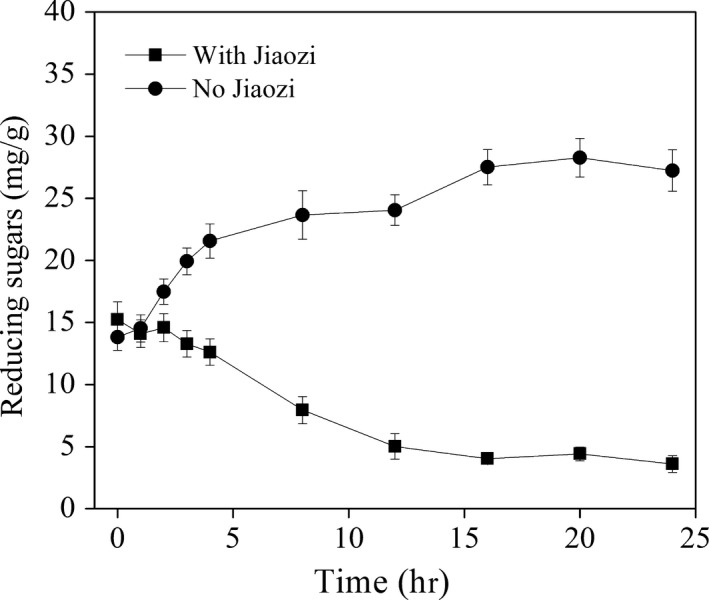
Evolution of reducing sugar during dough fermentation incubated with or without Jiaozi

The changes in the terms of reducing sugar during dough fermentation are shown in Figure 2. The results displayed that when Jiaozi was inoculated in the dough, the content of reducing sugar did not significantly change during the first 4 hr of fermentation, then gradually decreased until 12 hr, and after 12 hr, a fluctuation in the range from 3.6 to 5.0 mg/g dough was observed (Figure 2).

Changes of reducing sugar in the dough incubated with Jiaozi starter were an outcome of starch hydrolysis by amylases and carbohydrate consumption by the microorganisms in dough. This was hypothesized in the previous researches (Paramithiotis, Gioulatos, Tsakalidou, & Kalantzopoulos, 2006). For the uninoculated dough, the concentration of reducing sugar gradually increased during the spontaneous dough fermentation (Figure 2), which could be due to the continued hydrolysis of the starch fraction by endogenous flour amylases, but slower consumption by the indigenous microorganisms in the flour. The unchanged reducing sugar concentration in dough fermented by Jiaozi in the first 4 hr implies that the rate of starch hydrolysis by amylases and glucoamylase was almost equal to that of carbohydrate consumption by microbes. The results were quite different from yeast‐leavened dough where the significant decrease in reducing sugars with fermentation time was observed (Mustafa et al., 2009), which indicated the abundant carbon sources in the dough fermentation started by Jiaozi. The lack of competition among the microorganisms for carbohydrate could be beneficial to the stability of microbial community during dough fermentation and subsequently steamed bread quality and flavor. The hypothesis was in accordance with the previous study (Gobbetti, Corsetti, & Rossi, 1994).

### 3.3 Acidity changes and TTA analysis

**Figure 3:**
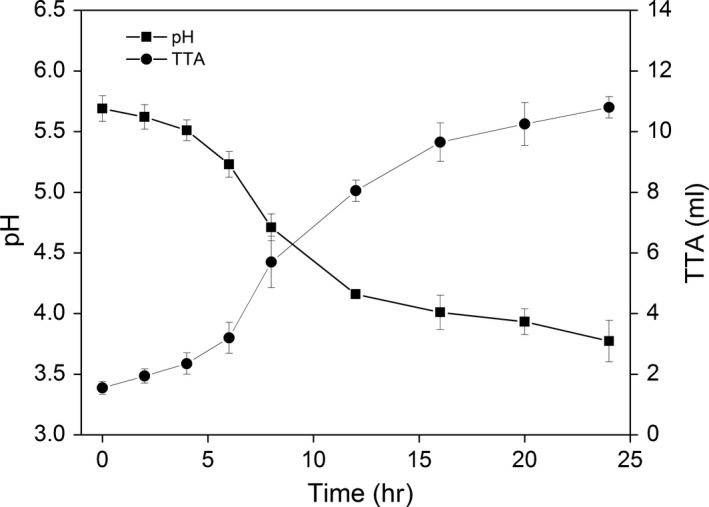
pH and TTA profiles of the dough fermented by the Jiaozi

The acidity analysis of the dough fermented by Jiaozi showed that in the beginning of the fermentation, the pH value of dough was 5.7 and slowly decreased in the first 4 hr (Figure 3). After 4 hr, the pH value rapidly dropped and stopped dropping at around 12 hr. Subsequently, the pH value slowly decreased. Correspondingly, the TTA accumulated as fermentation proceeded and it rapidly developed to 9.6 after 16 hr of fermentation (Figure 3). The values of pH and TTA were similar with the previous report of dough fermented by co‐culture of yeasts and LAB (Edema & Sanni, 2008). The dough became acidic and indicated that the acid‐producing bacteria gradually dominated the dough microflora and a large number of carbon sources were used to produce acid. Enumeration of numbers of microorganisms showed that the LAB number increased from 6.80 to 8.38 log CFU/g dough, whereas the yeast cell number only increased from 6.97 to 7.60 log CFU/g dough.

### 3.4 CO_2_ production and retention

**Figure 4:**
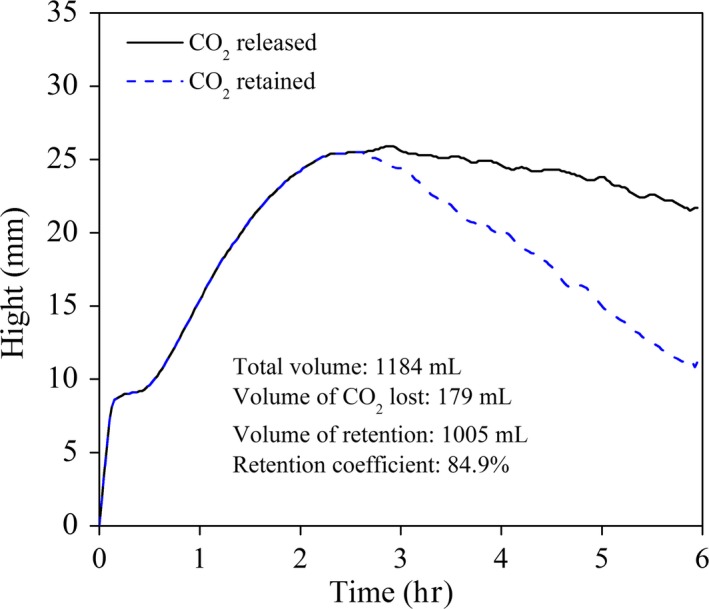
CO2 release and retention curves of dough inoculated with Jiaozi

The characteristics of CO_2_ production and retention of the dough started with Jiaozi are illustrated in Figure 4. The total volume of CO_2_ production increased rapidly after the addition of Jiaozi. The CO_2_ formation rate increased exponentially before running asymptotically toward zero in the first 2 h, and then, a relatively constant rate of CO_2_ production was observed (Figure 4). The CO_2_ production kinetics indicated that the yeast metabolism was activated gradually in the first 2 hr and high yeast activity was kept during the whole fermentation time or for a long time, indicating a stable fermentative capability.

The previous study has shown that gas formation rate is directly related to carbohydrates concentration and activity of yeast cells (Verheyen, Albrecht, Elgeti, Jekle, & Becker, 2015). It was impossible for CO_2_ formation rate to increased unrestrictedly due to the limitation of yeast metabolites potential and substrate availability. As above mentioned in Section 3.2, the substrate present in the dough was replete, and this might be one of the reasons of the deceased rate of CO_2_ production with the time. It has been reported that little oxygen is present in dough and is quickly exhausted shortly after kneading (Joye, Draganski, Delcour, & Ludescher, 2012), and yeast metabolism in dough is influenced by anaerobic conditions and only one third of CO_2_ is synthesized during the anaerobic fermentation compared with aerobic condition (Jayaram et al., 2013; Verheyen, Jekle, & Becker, 2014). Therefore, the results also indicated that the metabolism or fermentative activity of yeast in the dough could reach the maximum after 2 hr. Besides, the metabolism of other microorganisms could also affect the CO_2_ production of yeasts. This was in accordance with the previous report (Gobbetti et al., 1994).

The total CO_2_ produced was almost totally retained by dough in the first 3 hr (Figure 4), which indicated that the porosity did not formed (upper and lower lines were superimposed) and it was beneficial to gas retention. Even after fermenting for 6 hr, it also showed a significantly higher retention coefficient (84.9%). Lactic acid, mainly produced by LAB in Jiaozi, can partially account for the more elastic gluten structure (Gobbetti et al., 1994) and gave the high gas retention ability. Although a high level of gas production during fermentation is crucial for sufficient leavening of steamed bread, the volume might still be impaired if the produced CO_2_ cannot be stabilized during the production process.

### 3.5 Steamed bread quality evaluation

The effects of the fermentation method and time on the quality of steamed bread were evaluated. The results in Table 2 demonstrated that there was no significant difference in the specific volume, hardness, and sensory score between the steamed breads prepared by two‐stage method with 6 hr and 8 hr fermentation, respectively. Higher whiteness of the steamed bread prepared by two‐stage method with 6 hr fermentation was observed. A higher specific volume, whiteness, and sensory score of the steamed bread made by two‐stage method were obtained compared with that of one‐stage method.

**Table 2:** Quality of steamed breads prepared by Jiaozi

	Fermentation time (h)	Specific volume (ml/g)	Hardness (g)	Whiteness	Sensory score
Two‐stage method	6	2.35 ± 0.11^a^	2271 ± 150^a^	53.4 ± 0.31^a^	91 ± 5^a^
	8	2.41 ± 0.08^a^	2189 ± 161^a^	52.6 ± 0.35^b^	90 ± 8^a^
One‐stage method	1	1.93 ± 0.13^b^	5151 ± 301^b^	49.2 ± 0.53^c^	78 ± 7^b^

Means with different superscript letters within the same column are significantly different (*p < *0.05).

The specific volume, hardness, and sensory score of steamed bread are closely related to the CO_2_ production and metabolite by microorganisms and the gluten network structure of dough. The similar yeast activity and dough structure could be the reason for the comparable specific volume and hardness of steamed bread prepared by two‐stage method with 6‐hr and 8‐hr fermentation, respectively. A higher whiteness of steamed bread with 6‐hr dough fermentation might be related to the changed interior microstructure and metabolite influenced by the microbial activity (Li et al., 2015). When one‐stage method was used to ferment the dough, no porosity was formed and the gas cells could not well expanded and developed due to the lower gas production as described above, which could explain the unsatisfactory breadcrumb texture, lower‐specific volume, higher hardness, smaller compact gas cells, and denser crumb of the final product.

Based on the data shown in Table 2, the quality of the steamed bread prepared by two‐stage fermentation method was of better quality than the steamed bread prepared by one‐stage fermentation method. It should be noted that although the specific volume of steamed bread by one‐stage method using Jiaozi as starter was lower than that by two‐stage method, it still reached the national standard of China that the specific volume of the steamed bread is higher than 1.7 (SAC, 2007).

## 4 CONCLUSION

Some key factors involving in the dough fermentation process using Jiaozi starter were investigated. The results found that the predominant yeast and LAB species remained at a relatively constant ratio during the whole process of dough fermentation. The high rate of CO_2_ production with the fermentation time could be due to the high yeast activity for gas production as the carbohydrates were adequate during the first 8 hr of dough fermentation. Together with the quality evaluation of the final product, the results displayed the suitability and convenience of Jiaozi as starter in steamed bread production using a two‐stage procedure with flexible full fermentation periods. The work is helpful for the production procedure management and improvement of the quality and stability of final product in future.

## CONFLICT OF INTERESTS

The authors declare that they have no conflict of interest and the study does not involve any human or animal testing.
